# Real‐World Driving Data Indexes Dopaminergic Treatment Effects in Parkinson's Disease

**DOI:** 10.1002/mdc3.13803

**Published:** 2023-06-12

**Authors:** Jun Ha Chang, Danish Bhatti, Matthew Rizzo, Ergun Y. Uc, John Bertoni, Jennifer Merickel

**Affiliations:** ^1^ Department of Neurological Sciences University of Nebraska Medical Center Omaha Nebraska USA; ^2^ Department of Internal Medicine University of Central Florida Orlando Florida USA; ^3^ Department of Neurology University of Iowa Iowa City Iowa USA; ^4^ Neurology Service Iowa City VA Medical Center Iowa City Iowa USA

**Keywords:** Parkinson's disease, digital profiles, driving, dopaminergic medications, MDS‐UPDRS

## Abstract

**Background:**

Driving is a complex, everyday task that impacts patient agency, safety, mobility, social connections, and quality of life. Digital tools can provide comprehensive real‐world (RW) data on driver behavior in patients with Parkinson's disease (PD), providing critical data on disease status and treatment efficacy in the patient's own environment.

**Objective:**

This pilot study examined the use of driving data as a RW digital biomarker of PD symptom severity and dopaminergic therapy effectiveness.

**Methods:**

Naturalistic driving data (3974 drives) were collected for 1 month from 30 idiopathic PD drivers treated with dopaminergic medications. Prescriptions data were used to calculate levodopa equivalent daily dose (LEDD). The association between LEDD and driver mobility (number of drives) was assessed across PD severity, measured by the Movement Disorder Society Unified Parkinson's Disease Rating Scale (MDS‐UPDRS).

**Results:**

PD drivers with worse motor symptoms based on self‐report (Part II: *P* = 0.02) and clinical examination (Part III: *P* < 0.001) showed greater decrements in driver mobility. LEDD levels >400 mg/day were associated with higher driver mobility than those with worse PD symptoms (Part I: *P* = 0.02, Part II: *P* < 0.001, Part III: *P* < 0.001).

**Conclusions:**

Results suggest that comprehensive RW driving data on PD patients may index disease status and treatment effectiveness to improve patient symptoms, safety, mobility, and independence. Higher dopaminergic treatment may enhance safe driver mobility in PD patients with worse symptom severity.

Dopaminergic medications like levodopa reduce Parkinson's disease (PD) motor symptoms.^1^ They are typically prescribed or adjusted based on clinical exam and patient self‐report. However, effective PD medication adjustment can be difficult.^2^ Patient's self‐report may be unreliable because of reduced observational skills, bias, cognitive change, and lack of situational awareness.^3^, ^4^ Patients often act differently in controlled clinical settings than they do in their usual home settings. Because of these limitations, clinicians often lack continuous and objective data about how PD patient symptoms and treatment impact real‐world (RW) patient outcomes and daily activities. As a result, clinicians may underestimate patient impairments and treat symptoms less effectively, increasing risk of independence and mobility decline in PD. This has led to developing tools for remote patient monitoring (RPM) such as Personal KinetiGraph by global kinetics and others.^5^ Most efforts have focused on wearable devices such as watches or rings to monitor hand tremor and bradykinesia. Although in‐lab gait analyses have matured, no commercially available gait monitoring system for PD RPM exists.

Driving is a highly learned, complex task that is ubiquitous in modern society and a significant component of social mobility and independence. PD impairs motor and non‐motor abilities needed for safe driving.^6^ Although evidence does not suggest higher RW crash rates for PD compared to controls,^7^ drivers with PD show greater likelihood of crash in driving simulation and higher failure rate of on‐road driving tests.^7^, ^8^, ^9^ PD driver risk, safety errors, and errant driving behaviors likely increase with severe disease symptoms, advanced stage, and higher levels of functional impairments (motor and non‐motor [eg, cognitive and visual]).^10^, ^11^, ^12^, ^13^, ^14^, ^15^, ^16^ These factors encourage drivers with PD to regulate driving exposure (how often they drive) in general and in challenging driving environmental contexts (eg, night, long journey).^17^, ^18^, ^19^

Because dopaminergic medications alleviate motor symptoms for a period of time, PD drivers treated with the optimal medication dose may show greater mobility—regardless of disease severity. In turn, observing PD driver mobility may index PD severity and medication effectiveness.^17^, ^18^, ^20^, ^21^ We propose that digital biomarkers extracted from a patient's own typical driving behavior can provide continuous and objective RW data of PD symptom severity and medication effectiveness. Because patient behavior depends critically on the context and environment in which it is observed, RW data on PD patient behavior are needed to inform effective patient treatment. RW driving data show promise as a representative monitoring tool for most PD patients as >80% of PD patients are licensed drivers, and ~60% of them continue to drive for years after diagnosis.^22^

Digital driver health profiles can now be extracted from sensors readily available in current vehicle technology to index clinically relevant metrics of PD patient independence, quality of life, and RW functional impairments.^17^, ^18^, ^20^, ^21^ Our study builds on prior literature showing feasibility of mapping PD disease severity to digital vehicle data^18^ to determine if RW driving data can index PD medication management effectiveness. The goal of this pilot study is to evaluate the role of objective RW driving data as a digital biomarker for PD symptom severity and dopaminergic therapy effectiveness.

## Methods

### Study Design

**FIG. 1:**
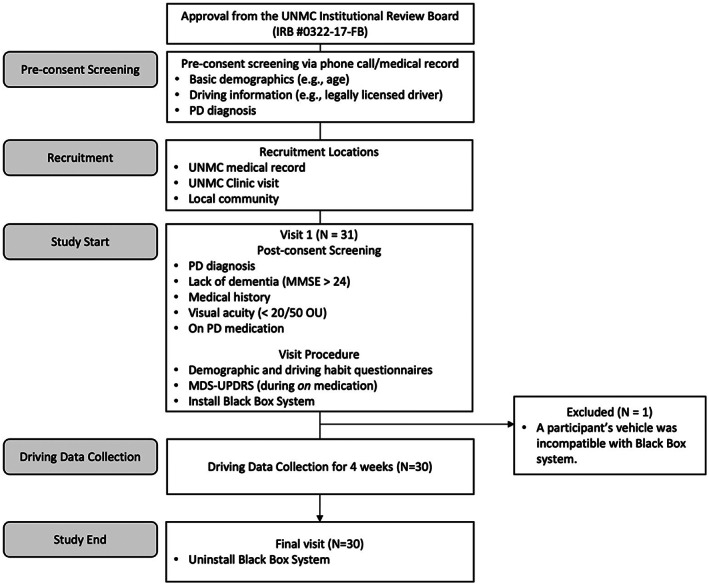
Flow chart of study design.

PD participants completed 4 weeks of continuous, RW driving data collection. Participants came to the study site at the start and end of the study period (Fig. 1). At the first visit, participants completed consent, laboratory assessments, and medical assessments. A driving data recording system was installed in the participant's personal vehicle and uninstalled at study end.

### Participants

Thirty‐one participants diagnosed with idiopathic PD were recruited from the University of Nebraska Medical Center (UNMC) Parkinson's disease clinic and local community in Nebraska at clinic visits.

Participants met United Kingdom Brain Bank Diagnostic Criteria,^23^ as assessed by a trained neurologist who specializes in movement disorders, and were treated with dopaminergic therapy for PD. All participants were active, legally licensed drivers who met Nebraska state licensure guidelines, including vision (<20/40 OU corrected or uncorrected). They were independently living drivers without dementia (based on medical records). Confounding medical conditions and medications were excluded. Excluded medical conditions were illness in the week before induction, neuropathy, severe pulmonary disease, congestive heart failure, major or active psychiatric disorders, non‐PD neurodegenerative disease, brain injury (stroke or traumatic brain injury), vestibular disease, sleep disorder, or non‐PD‐related mobility impairments. Patients on stimulants, sedating antihistamines, narcotics, anxiolytics, anticonvulsant, and major psychoactive medications were excluded. Participants consented to study participation following the UNMC Institutional Review Board guidelines (IRB 0322‐17‐FB). One participant was removed because of vehicle incompatibility with the driving data recording system. In total, 30 PD drivers completed the study. This provided 80% power to detect effects as small as 0.49 standard deviations (SDs) at a significance level of 0.05.

### Data Collection

#### Laboratory Assessments

Cognitive impairment and dementia were screened with the Mini‐Mental Status Exam [MMSE, score >24]^24^ and from medical records. Far/near visual acuity were assessed and screened with Early Treatment Diabetic Retinopathy vision chart.^25^ Medication history and usage were collected and screened via self‐report. All participants completed standardized, self‐reported questionnaires collecting demographics (age, sex, race, education, and socioeconomics), and driving habits (primary driving environment, driving experiences, and driving distance per week).

#### Medical Assessments

A neurologist specializing in movement disorders assessed each participant using the Movement Disorder Society Unified Parkinson's Disease Rating Scale (MDS‐UPDRS).^26^ The MDS‐UPDRS is a revised version of UPDRS that provides improved evaluation of various aspects of PD symptoms, including: Part (I) non‐motor experiences of daily living, Part (II) motor experiences of daily living, Part (III) motor examination, and Part (IV) motor complications. The assessment of MDS‐UPDRS for PD participants was made during on medication status.

Participants self‐reported all prescribed medications. Dopaminergic medications used to treat PD were used to calculate total levodopa equivalent daily dose (LEDD).^27^

#### Naturalistic Driving Assessment

A custom‐built sensor instrumentation system (Black Box) was installed at the study start in each participant's own vehicle to collect driving data. It continuously and passively recorded video (forward roadway, cabin), global positioning system (GPS), accelerometer, and on‐board diagnostic (engine throttle, rpm, and speed) data every second from on‐to off‐ ignition. The system was unobtrusively mounted on the forward windshield, next to the rearview mirror. Each driver was asked to drive as they typically would for the 4‐week observation period.

## Data Analysis

### Driver Mobility Outcome

Driver mobility was indexed as the total number of drives during the data collection period. We also analyzed driver mobility as a function of PD severity and dopaminergic medication intake to assess the effect of medication dosing on preservation of driving mobility as PD progresses.

### PD Disease Severity Covariates

Four subsections of MDS‐UPDRS were used to evaluate PD self‐reported and clinically assessed PD severity. Items contained in the subsections were rated with a scale from 0 to 4, with a higher score indicating more severe symptoms. Item scores were summed to produce four subscale scores. Subscale scores were converted to *z*‐scores and modeled as continuous predictors.

### LEDD Covariate

To standardize variable medication regimens, we computed total LEDD score to summarize total daily dopaminergic medication dosing.^27^ Table 1 shows PD treatment medications used in this sample and the number of participants using each medication. Almost all participants took Levodopa (n = 29), and some participants took other additional dopaminergic medications. The daily dose of each drug was multiplied using conversion factors validated in prior literature^27^, ^28^ and then summed to obtain total LEDD (Table 1). Each participant's total LEDDs were divided into two levels based on our sample characteristics to distribute evenly across categories participants and previous findings linking dyskinesia risk with the amount of total LEDD^29^; low (≤400 mg/day) and high (>400 mg/day). Twelve participants were assigned to the low LEDD category, and 18 participants were assigned to the high LEDD category (Table 2). Total LEDD category was entered in the model as a primary categorical predictor.

**TABLE 1:** A list of medications with number of participants and its conversion factor for levodopa equivalent daily dose

Medication	N	Conversion factor
Levodopa IR	29	1
Rasagiline	9	100
Amantadine	5	1
Ropinirole ER/IR	6	20
Selegiline	4	10
Pramipexole	3	100
Entacapone	1	0.33
Levodopa ER	1	0.5
Levodopa SR	1	0.75

Abbreviations: IR, immediate release; ER, extended release; SR, sustained release.

**TABLE 2:** PD patients’ demographic, self‐reported driving habits, and disease characteristics

Characteristics	PD (n = 30)
Demographics	
Age (years)	66.9 ± 6.5 (50–78)
Mean ± SD (range)	
Sex	
Male	21 (70%)
Female	9 (30%)
Education (years)	
Mean ± SD (range)	15.1 ± 1.80 (10–17)
Employment status	
Working	16 (53.3%)
Retired	14 (46.7%)
Season	
Winter	14 (46.7%)
Not‐winter	16 (53.3%)
Self‐reported driving habits	
Driving environment (primary)	
Rural	5 (83.3%)
Urban	25 (16.6%)
Driving experience (years)	
Mean ± SD (range)	51.1 ± 6.6 (35–62)
Driving per week (miles)	
Mean ± SD (range)	118 ± 83.0 (35–400)
PD disease‐specific measures	
Disease duration (years)	
Mean ± SD (range)	6.5 ± 3.9 (1–17)
Disease onset age (years)	
Mean ± SD (range)	60.5 ± 8.2 (40–76)
MDS‐UPDRS, mean ± SD (range)	
Part 1	5.7 ± 4.4 (0–22)
Part 2	7.3 ± 5.2 (0–25)
Part 3	34.4 ± 11.5 (13–61)
Part 4	3.5 ± 3.4 (0–13)
LEDD (mg/d)	
Overall, mean ± SD (range)	528.8 ± 204.4 (300–1077)
Low LEDD, mean ± SD (range)	350 ± 52.2 (300–400)
High LEDD, mean ± SD (range)	648 ± 178.6 (460–1077)
Hoehn and Yahr stage	
Median (IQR)	2 (2–2)

Abbreviations: PD, Parkinson's disease; SD, standard deviation; MDS‐UPDRS, Movement Disorder Society Unified Parkinson's Disease Rating Scale; LEDD, levodopa equivalent daily dose; IQR, interquartile range.

### Control Variables

Models were adjusted for control variables affecting driver behavior: age, sex (male vs. female), years of education, employment status (work vs. not working), and seasons (winter [November–March] vs. non‐winter [April–October]). Season controlled for overall variance in driving because of weather.^30^ All continuous variables were converted into *z*‐scores (eg, age, years of education).

### Analytic Plan

Descriptive statistics for demographic, self‐reported driving habits and behaviors, disease‐specific measures, and driver mobility were assessed to determine baseline driver characteristics. Pearson correlations were performed to examine general effects of control covariates on the average number of drives per day (total number of drives divided by the length of data collection in days) for continuous variables (age, years of education). Welch's two sample *t* tests were performed for categorical variables (sex, employment status, and seasons).

#### Modeling Approach

Our analytic goals were to determine whether PD symptom severity and the two level LEDD category were associated with driver mobility. Before modeling, we assessed the descriptive characteristics (eg, sample distribution) of each MDS‐UPDRS subscale score to determine appropriateness for modeling. After descriptive assessment, we conducted tests using Poisson regression models:

- Independent effects of disease severity on driver mobility: Each subscale score of MDS‐UPDRS was entered as a primary predictor in Poisson regression model with control covariates and an offset variable describing data collection in days.
- Independent effect of LEDD category on driver mobility: An identical Poisson regression model with the 2 level LEDD category (low [≤400 mg/day] and high [>400 mg/day]) as the primary predictor instead of MDS‐UPDRS subscale scores was performed.
- Interaction effect of disease severity depending on LEDD category on driver mobility: Interactions between the 2 level LEDD category and each MDS‐UPDRS subscale score were assessed using Poisson regression model, adjusting for data collection periods and control covariates. This resulted in three models with each combination of MDS‐UPDRS Part and LEDD category. Once significant interactions were confirmed, additional analyses were performed with data stratified by each level of LEDD category.

All *P* values were evaluated with 2‐sided alternative hypothesis tests at 5% significance.

## Results

### Demographic and Self‐Reported Driving Characteristics

Demographic and disease specific profiles of all participants (mean age = 66.9 years, range = 50–78 years) are shown in Table 2 including the male predominant sex distribution (male: n = 21; 70% of PD participants), which is typical of the PD population.^31^

### Disease Characteristics

Mean PD duration was 6.5 years (see Table 2). Mean LEDD was 528.8 mg/day. Participants whose disease duration was longer took, on average, higher doses of PD medications (*r* = 0.55, *P* < 0.01). MDS‐UPDRS Part I–III scores were typically mild‐to‐moderate, which is consistent with other PD driving studies.^32^, ^33^, ^34^ Part IV (medication side effects) scores were strongly skewed toward 0 (mean = 3.5, SD = 3.4, range = 0–13) with 33% of PD participants not reporting any motor complications (eg, dyskinesias, medication wear off). Because of limited range, we excluded Part IV from further analysis. Most participants fell in Hoehn and Yahr (H&Y) stage 2 or below (n = 23), consistent with the relatively early disease stage of most patients in our cohort.

### Naturalistic Driving Data

We collected 3974 drives (30,515 miles). On average each driver drove 133 drives (range = 66–301), with 4.2 drives each day (range = 1.6–9.4 drives) over 31.7 days (range = 24–56). Driver mobility was not significantly different across age (*r* = 0.01, *P* = 0.94), sex (*t* = 1.12, *P* = 0.29), education years (*r* = −0.21, *P* = 0.26), employment status (*t* = −0.26, *P* = 0.80), or season of driving (*t* = 1.71, *P* = 0.10). Table **S1** shows details on driver mobility across key sample demographics.

### Independent Effects of Disease Severity and LEDD on Driver Mobility

**FIG. 2:**
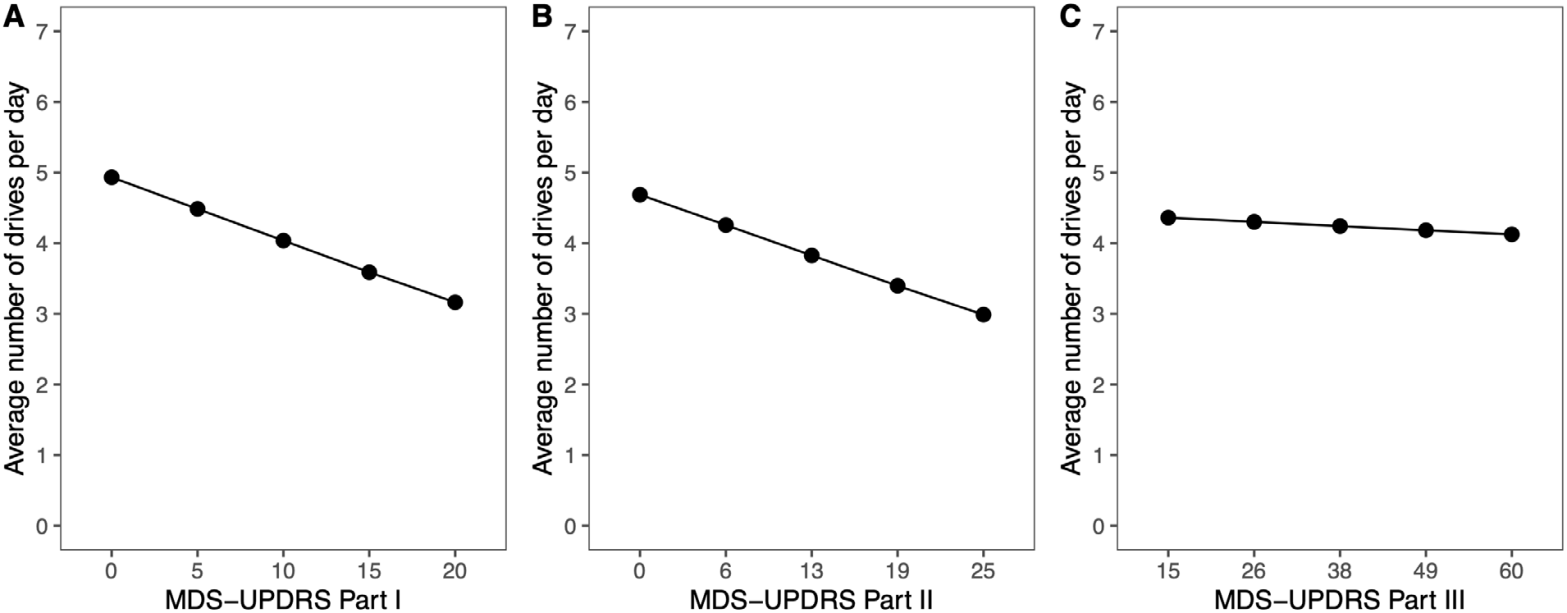
Disease severity measured in subscale scores of Movement Disorder Society Unified Parkinson's Disease Rating Scale (MDS‐UPDRS): Part I (**A**: non‐motor experiences of daily living), Part II (**B**: motor experiences of daily living), and Part III (**C**: motor examination) related to driver mobility.

MDS‐UPDRS and driver mobility showed significant associations (Fig. 2). PD drivers with worse symptoms in motor experiences of daily living (Part II) drove less often (Incidence Rate Ratio [IRR] = 0.96, 95% confidence interval [CI] = 0.92–0.99, *P* = 0.02). For each 1 SD (5.23) of Part II score increase, the number of drives decreased by 4%. A larger effect size was observed in Part III, where 1 SD of score increase resulted in a 10% reduction in number of drives (IRR = 0.90, 95% CI = 0.86–0.93, *P* < 0.001). Worsening symptoms of non‐motor experiences in daily living (Part I), however, were not associated with driver mobility (IRR = 0.98, 95% CI = 0.94–1.02, *P* = 0.28). In contrast, driver mobility was not different based on the high or low LEDD dosing category (cutoff of 400 mg, IRR = 0.98, 95% CI = 0.91–1.04, *P* = 0.50).

### Covariate Effect of Disease Severity and LEDD on Driver Mobility

**FIG. 3:**
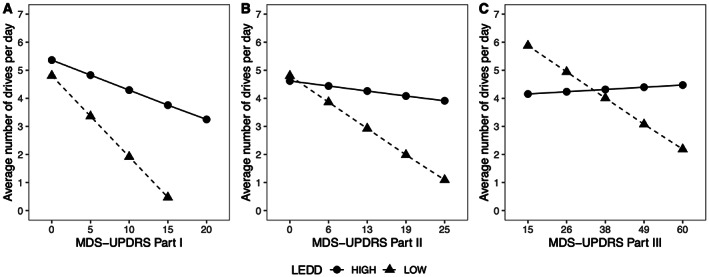
Driver mobility related to symptom severity in Movement Disorder Society Unified Parkinson's Disease Rating Scale (MDS‐UPDRS) Parts I (**A**), II (**B**), III (**C**) in context of levodopa equivalent daily dose (LEDD) dosing. A solid line with circles represents high LEDD and a dashed line with triangles represents low LEDD.

The impact of PD severity on driver mobility significantly varied depending on amount of LEDD dosing when comparing all three together (covariate analyses) (Fig. 3 and Table S2). We evaluated impact of MDS UPDRS Parts I, II, and III individually on driver mobility comparing high and low LEDD dosing (using a cutoff of 400 mg) across three models.

In Model 1 (Part I × LEDD categories, driver mobility), we found a significant interaction between LEDD category and MDS‐UPDRS Part I (IRR = 1.14, 95% CI = 1.023–1.26, *P* = 0.02). Our analysis demonstrated that for each 1 SD (4.45) increase of Part I score, the number of drives decreased significantly more for low LEDD group (23%) (IRR = 0.77, 95% CI = 0.66–0.89, *P* = 0.001), compared with high LEDD group (6%) (IRR = 0.94, 95% CI = 0.90–0.99, *P* = 0.03) (Fig. 3A). This indicates that increased dopaminergic medication dosing may preserve driver mobility as PD progresses and produces greater daily impairment from motor symptoms.

Similar interactions were observed in Part II (Model 2: IRR = 1.49, 95% CI = 1.37–1.62, *P* < 0.001) (Fig. 3B) and Part III (Model 3: IRR = 1.24, 95% CI = 1.15–1.34, *P* < 0.001) (Fig. 3C). As 1 SD of Part II score (5.23) increase, the number of drives decreased by 36% for low LEDD group (IRR = 0.64, 95% CI = 0.57–0.71, *P* < 0.001); however, no significant decrease was observed for the high LEDD group (IRR = 1.00, 95% CI = 0.95–1.05, *P* = 0.96). With a 1 SD of Part III score (11.5) increase, the number of drives decreased by 19% for the low LEDD group (IRR = 0.81, 95% CI = 0.75–0.87, *P* < 0.001) and decreased by 13% for the high LEDD group (IRR = 1.13, 95% CI = 1.07–1.20, *P* < 0.001).

In addition, across all three models, we found consistent effects of demographic covariates of age, education years, and seasons (Table S2). In general, older participants drove equally to younger drivers, but less educated drivers drove less than more educated drivers. Drivers drove overall less during winter than other seasons. Sex and employment status showed significant effects in some of the models. Male drivers drove less often than female drivers in Models 1 (Part I) and 2 (Part II), but this sex difference was not significant in Model 3 (Part III). Participants who were currently employed or working drove less than those who were not working in Model 2 (Part II), but the effect of employment status was not significant in Models 1 (Part I) and 3 (Part III).

## Discussion

This pilot study assessed the feasibility of using RW data from a driver's own vehicle as an objective digital biomarker of PD severity and the effectiveness of dopaminergic therapy. Patients treated with higher doses of dopamine (LEDD: >400 mg daily) showed greater driver mobility, despite worsening PD symptoms, than those receiving lower doses. The results demonstrate feasibility and utility to use driving as an objective tool to monitor PD patients’ daily routines and track RW effects of treatment on patient outcomes, like driver mobility.

In our study sample, we found that dopaminergic treatments improved driver mobility for motor and non‐motor PD symptoms, with motor symptoms showing larger improvements, in line with prior literature demonstrating a greater effect of dopaminergic medications on motor than non‐motor symptoms.^35^, ^36^ Clinicians must weigh the cost and benefits of increasing dopaminergic doses in patients against potential side effects. However, increased LEDD is associated with decreased impulse control,^37^ which might lead to less self‐regulation in driving.^38^ No patients in this sample carried a diagnosis of impulse control disorder.

Admittedly, our study sample is modest (n = 30), which may bias findings. Most of these active drivers with PD (n = 23) had H&Y 2 or lower, in line with the typical severity range of active PD drivers. Motor complications severity, which may be associated with greater rates of worse disease (and driving cessation), was not assessed in our analyses. Patients with severe cognitive impairments were not studied, likely restricting the opportunity to observe effects of cognitive decline on driver mobility in our sample. Nevertheless, in our stratified data analyses of medication dosing, drivers with more non‐motor decline drove less at high and low LEDD doses. A larger, less restricted sample in future research may improve the ability to consistently see these effects. Future research may also investigate how subtypes of PD (eg, motor dominant vs. postural instability and gait disorder), which may be more or less responsive to Levodopa, impact driver mobility patterns.^39^, ^40^ This may help disentangle the effect of dopaminergic dosing from driver mobility changes that may underlie these clinical presentations. Notwithstanding these limitations, we successfully replicated previous findings that worsening PD disease reduces driver mobility and achieved our primary research goal of demonstrating that RW driver digital health monitoring shows utility for informing PD treatment.

The present study shows the feasibility of using RW driving data to index the impact of symptom progression and dopaminergic treatments in PD even in the early stage of PD with relatively low dopaminergic therapy (average LEDD was 528.8 mg daily). Objective RW driving data in the PD drivers reported here spanned almost 4000 drives across 30,515 miles. Although this is a pilot study, the findings reveal important clinical considerations for healthcare providers working with the PD population. Ability to preserve daily mobility in PD patients is improved with greater symptom control. Although PD drivers with more severe symptoms avoided driving, they chose to drive more when receiving more dopaminergic medication. Because this judgment is based on objective driving data, reporting bias or inaccurate patient self‐reporting is minimized to better support clinical decisions. Daily driver mobility shows potential for digital biomarker development with further validation to index disease control and RW patient outcomes. Notably, it may function, with further development, as an objective measure for assessing the efficacy of interventions or disease‐modifying therapies to treat PD, as well as for monitoring and optimizing PD clinical care.

This study provides a roadmap for implementation of patient‐centered outcomes in PD using mobile digital health technologies.^41^ These data can be easily collected over long‐periods of time, permitting continuous evaluation of patient mobility, potential treatment effectiveness, and medication or symptom management. Building on the current study findings, future research can focus on the verification, technical, and clinical validation^42^ of naturalistic driving health data to track and detect the change of symptoms longitudinally. Future studies could also consider combining driving observation with medication tracking and using different RW driving variables (eg, distance driven) to understand how medication fluctuations impact daily driver mobility. Digital health monitoring from ubiquitous driving or other sensor data may provide egalitarian means to improve access to medical services for patients and caregivers who are physically isolated and geographically, culturally, or economically disadvantaged.

## Author Roles

(1) Research project: A. Conception, B. Organization, C. Execution; (2) Statistical Analysis: A. Design, B. Execution, C. Review and Critique; (3) Manuscript: A. Writing of the First Draft, B. Review and Critique.

J.C.: 2A, 2B, 3A

D.B.: 1C, 2C, 3B

M.R.: 1A, 1B, 1C, 2C, 3B

E.U.: 2C, 3B

J.B.: 1C, 2C, 3B

J.M.: 1A, 1B, 1C, 2C, 3B.

## Disclosures

**Ethical Compliance Statement:** This study was approved by UNMC Institutional Review Board guidelines (IRB No. 0322‐17‐ FB). Written informed consent was obtained from all participants. We confirm that we have read the journal's position on issues involving ethical publication and affirm that this work is consistent with those guidelines.

**Funding Sources and Conflicts of Interest:** The study was funded by the University of Nebraska Medical Center's Foundation (Stake‐a‐thon for Parkinson's). The authors declare that there are no conflicts of interest relevant to this work.

**Financial Disclosures for the Previous 12 Months:** E.Y.U. has been funded by National Institutes of Health, Veterans Administration, Department of Defense, Parkinson's Foundation, Patient Centered Outcome Research Institute via Parkinson's Foundation and has private compensations from honoraria for lecturing for Parkinson's Foundation and from Elsevier as associate editor of Parkinsonism and Related Disorders. Other authors declare no additional disclosures to report.

## Supporting information


**Table S1.** PD patients’ average number of drives per day across categorical demographics.
**Table S2.** Results of Poisson regression models for control covariates.

## References

[1] Poewe W , Seppi K , Tanner CM , et al. Parkinson disease. Nat Rev Dis Primers 2017;3:1–21. 10.1038/nrdp.2017.13 28332488

[2] Laditka JN , Laditka SB , Probst JC . Health care access in rural areas: evidence that hospitalization for ambulatory care‐sensitive conditions in the United States may increase with the level of rurality. Health Place 2009;15:761–770. 19211295 10.1016/j.healthplace.2008.12.007

[3] Marras C , Beck J , Bower J , et al. Prevalence of Parkinson's disease across North America. NPJ Parkinson's Disease 2018;4:1–7. 10.1038/s41531-018-0058-0 PMC6039505 30003140

[4] Pagan FL . Improving outcomes through early diagnosis of Parkinson's disease. Am J Manag Care 2012;18:S176–S182. 23039866

[5] Nahab FB , Abu‐Hussain H , Moreno L . Evaluation of clinical utility of the personal KinetiGraph® in the management of Parkinson disease. Adv in Parkinson's Dis 2019;8:42–61.

[6] Ergun Y , Uc MR . Driving and neurodegenerative diseases. Curr Neurol Neurosci Rep 2008;8:377–383. 18713573 10.1007/s11910-008-0059-1 PMC3097428

[7] Thompson T , Poulter D , Miles C , et al. Driving impairment and crash risk in Parkinson disease: a systematic review and meta‐analysis. Neurology 2018;91:e906–e916. 30076275 10.1212/WNL.0000000000006132

[8] Uc EY , Rizzo M , Anderson SW , Dastrup E , Sparks JD , Dawson JD . Driving under low‐contrast visibility conditions in Parkinson disease. Neurology 2009;73:1103–1110. 19805726 10.1212/WNL.0b013e3181bacf6e PMC2764395

[9] Devos H , Vandenberghe W , Tant M , Akinwuntan AE , de Weerdt W , Nieuwboer A , Uc EY . Driving and off‐road impairments underlying failure on road testing in Parkinson's disease. Mov Disord 2013;28:1949–1956. 24166984 10.1002/mds.25701

[10] Devos H , Vandenberghe W , Nieuwboer A , Tant M , Baten G , De Weerdt W . Predictors of fitness to drive in people with Parkinson disease. Neurology 2007;69:1434–1441. 17909156 10.1212/01.wnl.0000277640.58685.fc

[11] Uc EY , Rizzo M , Anderson SW , Sparks J , Rodnitzky RL , Dawson JD . Impaired visual search in drivers with Parkinson's disease. Ann Neurol 2006;60:407–413. 16969860 10.1002/ana.20958

[12] Uc EY , Rizzo M , Anderson SW , Sparks JD , Rodnitzky RL , Dawson JD . Driving with distraction in Parkinson disease. Neurology 2006;67:1774–1780. 17130409 10.1212/01.wnl.0000245086.32787.61

[13] Uc EY , Rizzo M , Anderson SW , Sparks JD , Rodnitzky RL , Dawson JD . Impaired navigation in drivers with Parkinson's disease. Brain 2007;130:2433–2440. 17686809 10.1093/brain/awm178

[14] Uc EY , Rizzo M , Johnson AM , Dastrup E , Anderson SW , Dawson JD . Road safety in drivers with Parkinson disease. Neurology 2009;73:2112–2119. 20018639 10.1212/WNL.0b013e3181c67b77 PMC2790224

[15] Rizzo M , Uc EY , Dawson J , Anderson S , Rodnitzky R . Driving difficulties in Parkinson's disease. Mov Disord 2010;25(Suppl 1):S136–S140. 20187237 10.1002/mds.22791 PMC3108508

[16] Uc EY , Rizzo M , O'Shea AMJ , Anderson SW , Dawson JD . Longitudinal decline of driving safety in Parkinson disease. Neurology 2017;89:1951–1958. 29021353 10.1212/WNL.0000000000004629 PMC5679414

[17] Stolwyk RJ , Scally KA , Charlton JL , Bradshaw JL , Iansek R , Georgiou‐Karistianis N . Self‐regulation of driving behavior in people with Parkinson disease. Cogn Behav Neurol 2015;28:80–91. 26102998 10.1097/WNN.0000000000000058

[18] Crizzle AM , Myers AM , Roy EA , Almeida QJ . Drivers with Parkinson's disease: are the symptoms of PD associated with restricted driving practices? J Neurol 2013;260:2562–2568. 23821027 10.1007/s00415-013-7017-9

[19] Brock P , Oates LL , Gray WK , et al. Driving and Parkinson's disease: a survey of the Patient's perspective. J Parkinsons Dis 2021;12:1–7. 10.3233/JPD-212686 34542030

[20] Crizzle AM , Myers AM . Examination of naturalistic driving pratices in drivers with Parkinson's disease compared to age and gender‐matched controls. Accid Anal Prev 2013;50:724–731. 22795036 10.1016/j.aap.2012.06.025

[21] Cordell R , Lee HC , Granger A , Vieira B , Lee AH . Driving assessment in Parkinson's disease—a novel predictor of performance? Mov Disord 2008;23:1217–1222. 18528878 10.1002/mds.21762

[22] Meindorfner C , Körner Y , Möller JC , Stiasny‐Kolster K , Oertel WH , Krüger HP . Driving in Parkinson's disease: mobility, accidents, and sudden onset of sleep at the wheel. Mov Disorder 2005;20:832–842. 10.1002/mds.20412 15726539

[23] Hughes AJ , Daniel SE , Kilford L , Lees AJ . Accuracy of clinical diagnosis of idiopathic Parkinson's disease: a clinico‐pathological study of 100 cases. J Neurol Neurosurg Psychiatry 1992;55:181–184. 1564476 10.1136/jnnp.55.3.181 PMC1014720

[24] Kochhann R , Varela JS , Lisboa CSM , Chaves MLF . The mini mental state examination: review of cutoff points adjusted for schooling in a large southern Brazilian sample. Dementia & Neuropsychologia 2010;4:35–41. 29213658 10.1590/S1980-57642010DN40100006 PMC5619528

[25] Group ETDRSR . Early treatment diabetic retinopathy study design and baseline patient characteristics: ETDRS report number 7. Ophthalmology 1991;98:741–756. 2062510 10.1016/s0161-6420(13)38009-9

[26] Goetz CG , Tilley BC , Shaftman SR , et al. Movement Disorder Society‐sponsored revision of the unified Parkinson's disease rating scale (MDS‐UPDRS): scale presentation and clinimetric testing results. Mov Disorder 2008;23:2129–2170. 10.1002/mds.22340 19025984

[27] Tomlinson CL , Stowe R , Patel S , Rick C , Gray R , Clarke CE . Systematic review of levodopa dose equivalency reporting in Parkinson's disease. Mov Disord 2010;25:2649–2653. 21069833 10.1002/mds.23429

[28] Schade S , Mollenhauer B , Trenkwalder C . Levodopa equivalent dose conversion factors: an updated proposal including Opicapone and safinamide. Mov Disord Clin Pract 2020;7:343–345. 32258239 10.1002/mdc3.12921 PMC7111582

[29] Warren Olanow C , Kieburtz K , Rascol O , et al. Factors predictive of the development of levodopa‐induced dyskinesia and wearing‐off in Parkinson's disease. Mov Disord 2013;28:1064–1071. 23630119 10.1002/mds.25364

[30] Mikuls TR , Merickel J , Gwon Y , et al. Vehicle control as a measure of real‐world driving performance in patients with rheumatoid arthritis. Arthritis Care Res (Hoboken) 2021;75:252–259. 10.1002/acr.24769 PMC8847538 34397172

[31] Wooten G , Currie L , Bovbjerg V , Lee J , Patrie J . Are men at greater risk for Parkinson's disease than women? J Neurol Neurosurg Psychiatry 2004;75:637–639. 15026515 10.1136/jnnp.2003.020982 PMC1739032

[32] Martínez‐Martín P , Rodríguez‐Blázquez C , Mario A , et al. Parkinson's disease severity levels and MDS‐unified Parkinson's disease rating scale. Parkinsonism Relat Disord 2015;21:50–54. 25466406 10.1016/j.parkreldis.2014.10.026

[33] Uc EY , Rizzo M , Johnson A , et al. Real‐life driving outcomes in Parkinson disease. Neurology 2011;76:1894–1902. 21624988 10.1212/WNL.0b013e31821d74fa PMC3115811

[34] Cubo E , Martinez Martin P , Gonzalez M , et al. What contributes to driving ability in Parkinson's disease. Disabil Rehabil 2010;32:374–378. 19958153 10.3109/09638280903168507

[35] Rinaldi D , Assogna F , Sforza M , Tagliente S , Pontieri FE . Rasagiline for dysexecutive symptoms during wearing‐off in Parkinson's disease: a pilot study. Neurol Sci 2018;39:141–143. 10.1007/s10072-017-3123-2 28956175

[36] Roy MA , Doiron M , Talon‐Croteau J , Dupre N , Simard M . Effects of Antiparkinson medication on cognition in Parkinson's disease: a systematic review. Can J Neurol Sci 2018;45:375–404. 29747716 10.1017/cjn.2018.21

[37] Corvol JC , Artaud F , Cormier‐Dequaire F , et al. Longitudinal analysis of impulse control disorders in Parkinson disease. Neurology 2018;91:e189–e201. 29925549 10.1212/WNL.0000000000005816 PMC6059034

[38] Merickel J , High R , Dawson J , Rizzo M . Real‐world risk exposure in older drivers with cognitive and visual dysfunction. Traffic Inj Prev 2019;20:S110–S115. 31821019 10.1080/15389588.2019.1688794 PMC7035173

[39] Mohl B , Berman BD , Shelton E , Tanabe J . Levodopa response differs in Parkinson's motor subtypes: a task‐based effective connectivity study. J Comp Neurol 2017;525:2192–2201. 28256710 10.1002/cne.24197 PMC6301039

[40] Vu TC , Nutt JG , Holford NH . Progression of motor and nonmotor features of Parkinson's disease and their response to treatment. Br J Clin Pharmacol 2012;74:267–283. 22283961 10.1111/j.1365-2125.2012.04192.x PMC3630747

[41] Espay AJ , Bonato P , Nahab FB , et al. Technology in Parkinson's disease: challenges and opportunities. Mov Disord 2016;31:1272–1282. 27125836 10.1002/mds.26642 PMC5014594

[42] Goldsack JC , Coravos A , Bakker JP , et al. Verification, analytical validation, and clinical validation (V3): the foundation of determining fit‐for‐purpose for biometric monitoring technologies (BioMeTs). npj digital Medicine 2020;3:1–15. 32337371 10.1038/s41746-020-0260-4 PMC7156507

