# Supplementary material for: Real‐World Driving Data Indexes Dopaminergic Treatment Effects in Parkinson's Disease
Source: Mov Disord Clin Pract. 2023 Jun 12;10(9):1324–32. doi: 10.1002/mdc3.13803 (PMC10525064; doi:10.1002/mdc3.13803)
Supplement: Supplementary file 1 — Table S1. PD patients’ average number of drives per day across categorical demographics. Table S2. Results of Poisson regression models for control covariates. [file MDC3-10-1324-s001.pdf]

# Supplemental Table 1

Supplemental Table 1
PD patients' average number of drives per day across categorical demographics.

| **Demographic Characteristics** | **Average Number of Drives per Day Mean ± SD (range) in miles** |
|---|---|
| Overall | 4.17 ± 1.61 (1.57 – 9.40) |
| Gender | |
| Male | 3.99 ± 1.31 (1.58 – 6.00) |
| Female | 4.59 ± 2.19 (2.55 – 9.40) |
| Employment status | |
| Working | 4.09 ± 1.20 (1.57 – 5.65) |
| Not-working | 4.24 ± 1.93 (2.12 – 9.40) |
| Season | |
| Winter | 3.65 ± 1.44 (1.57 – 6.75) |
| Non-winter | 4.63 ± 1.65 (2.20 – 9.40) |

# Supplemental Table 2

Supplemental Table 2
Results of Poisson regression models for control covariates

| | **Model 1 (MDS-UPDRS Part I x LEDD)** | | | **Model 2 (MDS-UPDRS Part II x LEDD)** | | | **Model 3 (MDS-UPDRS Part III x LEDD)** | | |
|---|---|---|---|---|---|---|---|---|---|
| | ***IRR*** | ***95% CI*** | ***p-value*** | ***IRR*** | ***95% CI*** | ***p-value*** | ***IRR*** | ***95% CI*** | ***p-value*** |
| Age | 0.975 | 0.939 – 1.012 | 0.183 | 1.012 | 0.976 – 1.050 | 0.513 | 1.032 | 0.994 – 1.073 | 0.100 |
| Gender (Male) | 0.790 | 0.729 – 0.856 | < 0.001 | 0.898 | 0.835 – 0.965 | 0.003 | 0.935 | 0.864 – 1.011 | 0.090 |
| Education (years) | 0.922 | 0.889 – 0.957 | < 0.001 | 0.934 | 0.898 – 0.970 | < 0.001 | 0.936 | 0.899 – 0.974 | 0.001 |
| Season | 0.915 | 0.843 – 0.992 | 0.031 | 0.840 | 0.782 – 0.902 | < 0.001 | 0.875 | 0.818 – 0.936 | < 0.001 |
| Employment (working) | 0.975 | 0.905 -1.049 | 0.493 | 0.901 | 0.835 – 0.973 | 0.008 | 0.975 | 0.908 – 1.047 | 0.487 |
